# First-In-Human Trials of GamTBvac, a Recombinant Subunit Tuberculosis Vaccine Candidate: Safety and Immunogenicity Assessment

**DOI:** 10.3390/vaccines7040166

**Published:** 2019-11-01

**Authors:** Daria V. Vasina, Denis A. Kleymenov, Victor A. Manuylov, Elena P. Mazunina, Egor Yu. Koptev, Elena A. Tukhovskaya, Arkady N. Murashev, Alexander L. Gintsburg, Vladimir A. Gushchin, Artem P. Tkachuk

**Affiliations:** 1 N.F. Gamaleya Federal Research Centre for Epidemiology and Microbiology, Ministry of Health of the Russian Federation, 123098 Moscow, Russia; d.v.vasina@gmail.com (D.V.V.); 10000let@rambler.ru (D.A.K.); victormanuilov@yandex.ru (V.A.M.); naldinaelena@rambler.ru (E.P.M.); egor.koptev@gmail.com (E.Y.K.); gintsburg@gamaleya.org (A.L.G.); artem.p.tkachuk@gmail.com (A.P.T.); 2 Branch of Shemyakin and Ovchinikov Institute of Bioorganic Chemistry, Russian Academy of Sciences, 142290 Pushchino, Russiamurashev@bibch.ru (A.N.M.); 3 Infectology Department, I. M. Sechenov First Moscow State Medical University, 119146 Moscow, Russia; 4 Department of Virology, Lomonosov Moscow State University, 119991 Moscow, Russia

**Keywords:** tuberculosis, subunit vaccine, BCG booster, clinical trials, safety and immunogenicity

## Abstract

Tuberculosis is known to be the biggest global health problem, causing the most deaths by a single infectious agent. Vaccine-development efforts are extremely important. This paper represents the results of the first-in-human trial of recombinant subunit tuberculosis vaccine GamTBvac in a Phase I study. GamTBvac is a new BCG booster candidate vaccine containing dextran-binding domain modified Ag85a and ESAT6-CFP10 MTB antigens and CpG ODN adjuvant, formulated with dextrans. Safety and immunogenicity of GamTBvac were estimated in an open-label clinical trial on 60 *Mycobacterium tuberculosis* uninfected (MTB-uninfected) volunteers previously-vaccinated with Bacillus Calmette—Guérin vaccine (BCG). The candidate vaccine had an acceptable safety profile and was well-tolerated. Three different vaccine doses with a double-immunization scheme were assessed for immunogenicity and induced a significant increase in IFN-γ in-house IGRA response and IgG ELISA analysis. Among them, the half dose vaccine group (containing DBD-ESAT6-CFP10, 12.5 μg; DBD-Ag85a, 12.5 μg; CpG (ODN 2216), 75 μg; DEAE-Dextran 500 kDa, 250 μg; and Dextran 500 kDa, 5 mg) provided high, early and stable in time immune response specific to both protein antigen fusions and is proposed for the further studies.

## 1. Introduction

Despite an intensive global fight against tuberculosis (TB), annual World Health Organization (WHO) reports do not suggest that we are on a secure way of solving this problem. Thus, the WHO has developed a global strategy and outlined targets for tuberculosis prevention, care, and control (WHO’s End TB Strategy) [1]. The current strategy implies the reduction of TB deaths by 95% and new cases by 90% when compared with 2015 data. It is estimated that optimization of existing prevention and treatment methods could reduce incidence by 10%, while the development of new approaches would lead to a reduction to the expected 90% level by 2025 [2]. Indeed, these estimates do not look realistic by the end of 2019; however, it does not mean that new TB vaccines are no longer needed.

In the long-term perspective, poverty and malnutrition eradication as well as improvement of social environment are important. Treatment of latently infected people to mitigate TB reservoirs might be effective in developed countries in Europe and North America, where the number of TB cases is relatively low [3]. However, in conditions of heterogeneous in-country morbidity ranging from 0 to 200 cases per 100,000 people, vaccination is the most sustainable way to control infection. Total vaccination by contemporary BCG strains does not solve the problem [4]. Thus, it is obvious that global control over this disease can be achieved through the creation of an effective number of prophylactic and therapeutic vaccines, the development of diagnostics, and the optimization of treatment regimens [5].

A significant proportion of vaccines currently being developed are recombinant preparations, including antigens of *Mycobacterium tuberculosis*, some of which are present in BCG [6,7]. In this case, these are called “booster vaccines” that are aimed at enhancing immunity generated by BCG and amount for primary immunity to antigens absent in BCG. Subunit recombinant vaccines are biotechnologically defined, and being mixed with appropriate antigens can be highly immunogenic in a wide group of people, and safe for use by HIV-positive individuals [8].

In Russia, tuberculosis is included in the list of socially significant diseases. Considerable attention has been paid to its prevention and diagnosis. The problem of multi-drug-resistant (MDR) tuberculosis spread is particularly acute in Russia. Thus, in total global incidence of MDR tuberculosis, the joint contribution of India, China, and Russia is estimated at 45% [3]. The overall incidence of tuberculosis in Russia itself remains quite high, averaging 60 per 100,000 inhabitants, and, in some regions, the figure reaches 160 cases; about half of the cases, 29 per 100,000, are MDR-TB [3].

Due to the urgent global need for TB vaccines and for improvement of the TB situation in Russia, recombinant subunit candidate vaccine GamTBvac was developed and investigated. The vaccine contains two *Mycobacterium tuberculosis* (MTB) antigens, Ag85a (MTB multistage secreted acyltransferase of antigen 85 complex) and ESAT6-CFP10 (the fusion of MTB early secreted antigenic target 6 kDa and the 10-kDa culture filtrate protein), fused with a dextran-binding domain (DBD) from *Leuconostoc mesenteroides* for noncovalent immobilization on dextran [9]. Dextran is known to be able to mediate both humoral and cell immunity [10]. The dextran 500 kDa polysaccharide was used for immobilization of the proteins Ag85a and ESAT6-CFP10, and the modified dextran DEAE 500 kDa with attached diethylaminoethyl polycation—for CpG oligonucleotides (TLR9 agonists). A mixture consisting of DEAE-dextran core covered with CpG oligodeoxynucleotides was used as vaccine adjuvant [11]. CpG ODNs of different classes are Toll-like receptor 9 (TLR9) agonists, able to activate an innate immune response through the improvement of antigen presentation and the induction of vaccine-specific responses. Recombinant antigens fused to a DBD bind to the dextran strongly, but not covalently, which provides constant and slow release of vaccine components from the carrier matrix due to its dissociation. This leads to a prolonged interaction of the components of the vaccine with the immune system, sufficient to induce a strong and stable immune response. On the other hand, the specific interaction of dextran binding domain with a matrix provides a high density of antigen incorporation, reduction of the vaccination volumes and reduces the likelihood of adverse events. GamTBvac preclinical studies showed high immunogenicity and protective efficacy in experimental murine and guinea-pig animal models [9]. Here, we report the results of a Phase I open-label clinical trial investigating the safety and immunogenicity of multi-subunit BCG booster candidate vaccine GamTBvac administered in MTB-uninfected BCG-immunized volunteers living in Russia (Moscow region).

## 2. Materials and Methods

### 2.1. Vaccine Production

Vaccine composition and production is described in details in the patent RU 2 665 817 C1 and [9]. In brief, two recombinant proteins, DBD-AG85a (MTB multistage secreted acyltransferase of antigen 85 complex) and DBD-ESAT6-CFP10 (the fusion of MTB early secreted antigenic target 6 kDa and the 10-kDa culture filtrate protein), were constructed and purified on the Butyl-Toyopearl hydrophobic column (Tosoh Bioscience LLC, King of Prussia, PA, USA), each coding for a chimeric gene composed of nucleotide sequence of DBD gene, Gly-Ser spacer and nucleotide sequence of either Ag85a or ESAT6-CFP10 MTB antigens. The vaccine was formulated with the adjuvant containing dextran 500 kDa (Dextran 500 Pharmaceutical Quality, Pharmacosmos, Denmark), dextran DEAE 500 kDa (DEAE-Dextran Pharmaceutical Quality, Pharmacosmos, Denmark) and CpG ODN 5′-ggGGGACGA:TCGTCgggggg-3′ (synthesized by the chemical group of the Laboratory of the Biologically Active Nanostructures at Gamaleya Federal Research Centre for Epidemiology and Microbiology). The final product (vaccine) was manufactured by Gamaleya Federal Research Centre for Epidemiology and Microbiology in an accredited GMP facility and supplied to the study site as a lyophilized product.

### 2.2. Study Design and Ethical Considerations

This is a Phase I, open-label, first-in-human clinical trial in BCG-vaccinated adults vaccinated with candidate multi-subunit BCG booster vaccine GamTBvac. The aim was to assess the safety and immunogenicity of GamTBvac in volunteers over the course of five months (140 days), as well as select the optimal dose of administration. The vaccine was administrated subcutaneously in accordance with the experimental plan on Days 0 and 57 (with the exception of Group 2 with a single injection). The trial was conducted in accordance with the Helsinki Declaration and Good Clinical Practices (ICH-GCP), and was externally monitored by an independently contracted research organization (Chromos Ltd., London, UK). The study was approved by the Council of Ethics at the Ministry of Health of the Russian Federation (extracted from Protocol No. 87, 26 August 2014; permission of the Ministry of Health of the Russian Federation to conduct clinical trial No. 179, 10 April 2015), and by the local ethics committee of the research center of I.M. Sechenov First Moscow State Medical University (extracted from Protocols No. 05–15, 20 May 2015, and No. 05–17, 14 June 2017). Written informed consent was obtained from all participants. This trial was registered on clinical-trial database ClinicalTrials.gov ID NCT03255278 [12].

### 2.3. Recruitment and Enrolment

Healthy adult volunteers, aged 18–49, were recruited from the general population of Moscow and the Moscow region, Russia. For inclusion, participants had to be generally healthy, HIV-negative, with no history of chronic medical conditions, and be BCG-immunized. Prior BCG immunization was determined by the presence of a characteristic scar, documentational approval of BCG vaccination in the volunteer medical history, and a reasonable reaction (3–9 mm) in response to tuberculin (Mantoux screening test). No pregnant or lactating women were included. Screening procedures included the collection of a personal medical history, a physical examination, chest radiography, blood collection for baseline chemistry and hematology, general urine analysis, and Hepatitis B and C serology. A QuantiFERON Gold-In-Tube test (QFT, Qiagen, Germantown, MD, USA) was used to detect MTB infections. Participants with negative QFT results were immunized with the GamTBvac.

We allocated the volunteers into five study groups (1:1:1:1:1) for subcutaneous vaccination. For the double-vaccination groups, the second administration of the vaccine was performed two months (Day 57) after the first administration. The research included five groups with 12 volunteers in each group (Table 1). This sample size was judged sufficient to determine the character and magnitude of the outcome measures, and especially serious and severe adverse events. Screening and participant numbers were assessed in chronological order.

### 2.4. Follow-Up and Safety Evaluation (Clinical Procedures)

After each vaccination, all vaccinees were observed for 24 h inpatient hospital. Afterwards, participants were evaluated on Days 1, 3, 7, 14, 42, 57, 63, 98, and 140 postvaccination. A physical examination was done at each visit. Blood for biochemistry and hematology tests, and urine for urinalysis were collected on Days 0 (screening and after vaccination), 1, 42, 57, 63, 98, and 140. Adverse events (AEs) were registered during the entire study by nurses, and their vaccine-relatedness was assessed by the study physician. Diary cards were given to participants to monitor possible adverse events after vaccination.

### 2.5. Immunogenicity Assays

Venous blood was collected for immunogenicity studies before immunization and on Days 1, 42, 63, 98, and 140. Immunogenicity assays included IgG antibody and cytokine multiplex immunology assay (MIA) MagPix (Luminex Corporation, Austin, TX, USA). An in-house interferon gamma release assay test (IGRA) was also used to assess IFN-γ expression after vaccination with GamTBvac. The assays were conducted as described previously [9,13]. In brief, 100 μL of whole blood was taken in a vacuum tube with lithium heparin (Vacuette TUBE, Greiner bio-one, Kremsmünster, Austria) that was supplied with 600 μL of growth medium (90% medium 199, 10% fetal bovine serum, 2 mM L-glutamine, 10 mM HEPES, 50 μg/mL gentamicin sulfate (PanEco, Moscow, Russia). Samples were separately stimulated with recombinant antigens DBD-Ag85a and DBD-ESAT6-CFP10 at a final concentration of 50 μg/mL. Concanavalin A from *Canavalia ensiformis* (Sigma-Aldrich, Taufkirchen, Germany) was used as a nonspecific inducer of IFN-γ production by lymphocytes for positive stimulation control. Samples without any stimulation were used as a negative control. Stimulation was conducted for 72 h under sterile humid conditions at 37 °C. Upon incubation, interferon gamma was quantified using an IFN-ELISA-BEST kit (А-8752, Vector-Best, Novosibirsk, Russia). Increased levels of interferon gamma as compared to levels of its spontaneous production in the negative control were regarded as a positive response to stimulation. The same samples were used for cytokine panel assessment with the MILLIPLEX MAP Human Cytokine/Chemokine Magnetic Bead Panel—Immunology Multiplex Assay KIT (HCYTOMAG-60K, Millipore, Danvers, MA, USA) according to the manufacturer’s recommendations.

For the serology-based quantification of antibodies to Ag85a, ESAT6, CFP10, and DBD, and their fusions, DBD-Ag85a and DBD-ESAT6-CFP10, 6 xMAP-based monoplex assays were used (Luminex Corporation, Northbrook, IL, USA). The optimal quantities of (fusion) antigens (5–20 μg per 10^6^ microspheres) were coupled to six microsphere sets through carbodiimide reactions according to a protocol described previously [13,14]. An indirect serological assay was run as recommended in [14]. Fifteen microliters of PBS-TBN (PBS, 0.1% BSA, 0.02% Tween-20, 0.05% NaN_3_) with 2500 microspheres per region and 50 μL of serum prediluted with PBS-TBN 50-fold (to a final dilution of 1:100) was placed into a well of a 96-well Microlon flat bottom clear polystyrene plate (Greiner, Nussbach, Austria). The mixture was incubated for 60 min at 25 °C and 800 rpm, and then washed using handheld magnetic separator MILLIPLEX (Merck Millipore, Darmstadt, Germany). Then, 100 μL PBS-TBN was added into each well, and the plate was left in the shaker for 30 s at 800 rpm for separation; two washing cycles were run (washing steps were the same throughout this part of the experiment). Then, microspheres were resuspended in 50 μL PBS-TBN and combined with 50 μL of 5 μg/mL anti-human IgG goat antibodies conjugated with phycoerythrin (One Lambda/ThermoFisher Scientific, Waltham, MA, USA). The final dilution of the conjugate in each well was 2.5 μg/mL. The suspension was incubated in a thermoshaker for 30 min at 25 °C and 800 rpm, and washed. The washed microspheres were resuspended in 100 μL PBS-TBN. Results were processed using MAGPIX (Luminex, Northbrook, IL, USA). For analysis, a minimum of 100 microspheres of the same region per well were used. Results of antibody quantification were expressed as median fluorescence intensity (MFI). For each sample, the negative control (normal rabbit serum) was subtracted from the raw value. 

### 2.6. Statistical Analysis

Statistical tests were performed with Prism (GraphPad, San Diego, CA, USA). Nonparametric tests were used as the method of choice. Multiple comparisons were performed with Kruskal–Wallis tests, with Dunn’s Multiple-Comparison Test. The primary study outcome was safety, as assessed by the frequency and severity of vaccine-related local and systemic AEs. Safety data were summarized by the frequency and severity of AEs using descriptive statistics. The Kruskal–Wallis test was used to determine differences between groups.

The secondary study outcome was immunogenicity. MIA, antibody-response (ELISA), and IFN-γ release-assay (IGRA) statistical analyses were performed using GraphPad Prism. The Wilcoxon matched-pairs signed rank test was used to detect differences between time points in the same group.

## 3. Results

### 3.1. Participants

**Figure 1:**
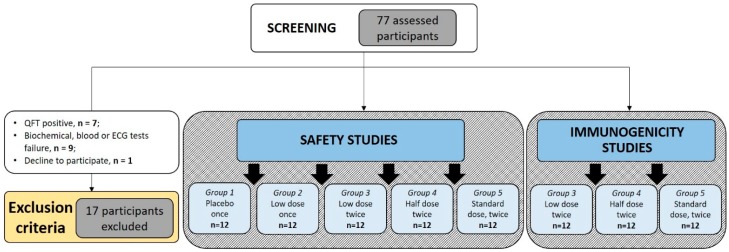
Study flow-path. Diagram of participants assessed for eligibility, enrolled, and vaccinated. Demographic details of individuals allocated in five study groups are shown in Table 2.

Seventy-seven volunteers were screened between September 2014 and December 2017. During the screening period, a total of 16 volunteers were excluded due to noncompliance with the inclusion criteria of the study, and one volunteer declined participation due to personal reasons. Seven participants were excluded because of positive QTF results (about 10%). Reasons for the exclusion of other volunteers are given in Figure 1. Sixty participants were recruited for the investigation and allocated to five study groups (Figure 1).

Participants in all groups were of European descent. Analysis of age and gender distribution showed significant age differences between Groups 3–5 and Group 1 (*p* = 0.008, *p* = 0.011, and *p* = 0.008, correspondingly). Group 2 included significantly more male volunteers (66.67%). Group 3 in general contained older people (*p* = 0.008). The shift in gender and age structure could be explained by the absence of general randomization between Groups 1 and 2, and among Groups 3–5. During the first stage, Group 1 and 2 volunteers were randomized. During the second stage, one year later, Group 3–5 participants were independently randomized. Such a design allowed assessment of single-immunization vaccine safety before implementation of double immunization and dosing the escalation protocol (Table 1). Once they entered the study, all participants received the appropriate vaccination according to the study design (Figure 1).

### 3.2. Vaccine Safety

Vaccine safety was assessed for 60 volunteers (Groups 1–5). Safety assessment was carried out with the onset of adverse events [15] observed 140 days after administration of the vaccine.

There were no clinically significant changes in any of the monitored parameters, including blood biochemistry or hematology for any of the subjects vaccinated with GamTBvac. Sixty adverse events were recorded in total (Table 3). All adverse events were mild in severity; six cases were estimated by the researchers as unrelated to the vaccination and 54 as possibly related to the vaccination. The most common AEs were development of erythema in the site of injection (38 cases), C-reactive protein increase (five cases), blood creatine phosphokinase increase (four) and red-blood-cell sedimentation-rate increase (two). Only one volunteer’s temperature rose following the vaccination in Group 5. All related AEs and their severity are shown in Table 3. The identified deviations of laboratory parameters and electrocardiogram (ECG) parameters were regarded by researchers of the certified clinical center as clinically insignificant [14].

In Groups 2–5, development of hyperemia at the injection site was reported within 24 h postvaccination for 6, 10, 12, and 12 volunteers, respectively (Table 3). In the case of Group 5, two cases of hyperemia out of twelve were registered after the second GamTBvac administration on Day 57. In all cases, the lesion was round, with the size varying 1.5–5.5 cm after the first injection, and 1.0–1.5 cm after revaccination, with no rise above the skin surface, and was painless on palpation. Development of the reaction stopped on its own within three days without special treatment.

We observed significant differences between the number of adverse events in the single and double vaccine administration, as well as in the groups with dose escalation. The highest number of AEs was observed in Groups 4 (21 AEs in 12/12 (100%) volunteers) and 5 (17 AEs in 12/12 (100%) volunteers). Among the most frequent AEs were C-reactive protein (CRP) increases. There were five cases of CRP increase: in Group 3, single increase in one of the volunteers was up to 1.88 mg/L on Day 42; in Group 4, up to 11 mg/L at Day 98, up to 20 mg/L at Day 98 and up to 57 mg/L at Day 63 in three different volunteers; and, in Group 5, up to 23 mg/L at Day 1 (24 h after the first vaccination). All cases were designated as mild, with a recovery outcome for all volunteers. CRP serves as an early marker of inflammation in an acute-phase of infection process. During the inflammation states, CRP levels rise rapidly within the first 6–8 h with a peak after 48 h. Only for Group 5 the early CRP increase was observed within first days postvaccination.

Thus, two AEs were assessed as possibly related to vaccination and graded as mild, both in Group 5: increase of C-reactive protein (up to 23 mg/L), and body temperature increase up to 38.0 °C (12 h postvaccination). All AEs were transient and resolved without the use of any concomitant therapy. Thus, an acceptable level of GamTBvac safety could be concluded for all doses.

### 3.3. Vaccine Immunogenicity

#### 3.3.1. Antigen-Specific IFN-γ Response

Since the administration of the GamTBvac candidate vaccine involves double administration with a two-month interval, vaccine immunogenicity was studied with all 36 volunteers corresponding to Groups 3–5. Humoral (IgG) and cell-mediated (IGRA IFN and cytokine panel) immunogenicity were investigated.

Formation of an antigen-specific T-cell response to antigens included in the GamTBvac was estimated by IFN-γ level change before and after blood stimulation with DBD-ESAT6-CFP10 and DBD-Ag85a. The dynamics of cytokine secretion was investigated on Days 1, 42, 63, 98, and 140.

**Figure 2:**
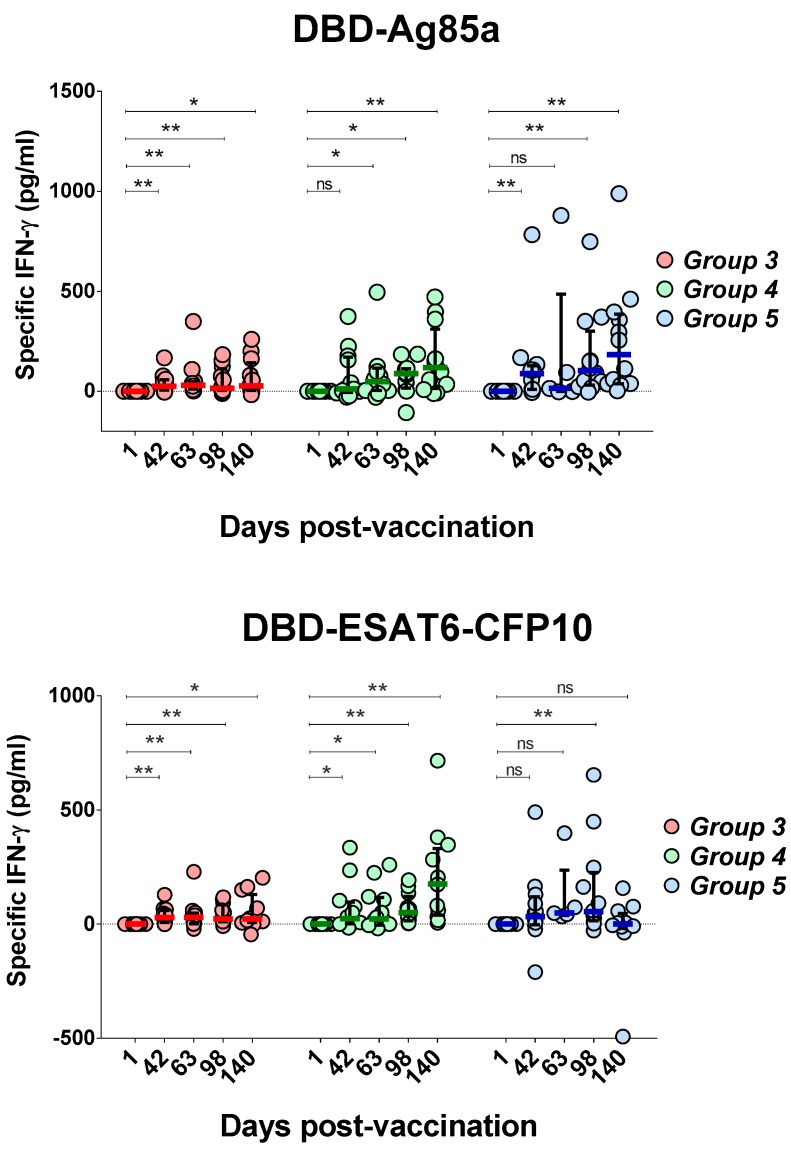
Changes of interferon gamma (IFN-γ) production after whole-blood stimulation in three groups under investigation. IFN-γ levels specific to both vaccine proteins were measured on five study days, beginning with first day after first vaccination as a baseline. Medians and interquartile range (IQT) are shown. Baseline indicated with dotted line. Difference in medians was not statistically significant between groups (DBD-Ag85a overall *p* = 0.0873; DBD-ESAT6-CFP10 overall *p* = 0.3165). Wilcoxon matched-pairs signed rank test was used to detect differences between time points in the same group. * *p* < 0.05, ** *p* < 0.01.

Both antigens activated cytokine production and contributed to the formation of durable cellular immunity after GamTBvac immunization (Figure 2) up to five months following vaccination. 

An increase in the production of IFN-γ in response to stimulation with the DBD-Ag85a antigen was first noted on Day 42, before the second immunization in Groups 3 (*p* = 0.0024) and 5 (*p* = 0.0024) (Figure 2). For Group 4, the mean values of IFN-γ production also increased at Day 42, but the observed differences were not statistically significant (*p* = 0.0771). Overall, in Groups 4 (half dose) and 5 (full dose), pronounced and significant differences were observed compared to low-dose administration (Group 3). Up to 98 days of observation, at any time point postvaccination, a full dose of the vaccine (Group 5) yielded higher levels of median IFN-γ induction. However, differences between Groups 4 and 5 ceased to be significant at the last observation point.

Similar dynamics of IFN-γ production was observed with DBD-ESAT6-CFP10 antigen blood stimulation. An increase in IFN-γ levels was observed as early as Day 42 in Groups 3 (*p* = 0.001) and 4 (*p* = 0.0122). For Group 5, the increase at Day 42 was not statistically significant (*p* = 0.1099). In Group 5, lower immunogenicity was observed compared to Groups 3 and 4. In one case out of 12 for the DBD-ESAT6-CFP10 antigen, there was significant decrease in the interferon response on Day 140 of observation. Generally, with a low (Group 3) and half dose (Group 4), a more uniform immune response was observed.

Taking into account the immunogenicity of both antigens, only Group 4, with a half vaccine, showed a sustainable immune response. Both vaccine antigens stimulated a persisting (up to 140 days) cellular immune response. Using the half dose of GamTBvac, we did not observe a drop in IFN-γ production by the end of the study. An early T-cell response stimulated by the vaccination could be protective, but could also be exhausted during the course of an MTB infection [16,17]. Thus, our data allowed to propose prolonged action of the candidate vaccine.

#### 3.3.2. Cytokine Profiling of T-Cell Response

Although the concept of Th1-type vaccine-induced immunity for protection against TB is prevalent, the development of vaccines that enhance other types of immune response is also of the current scientific interest. To investigate the direction of cell differentiation into one of the Th types of response after GamTBvac administration, we assessed the specific cytokine levels for the half-dose (Group 4) vaccine administration, as it possessed a concordant IFN-γ stimulation effect in the studied groups. The whole-blood samples were stimulated by antigens and assayed for cytokines (IFN-γ, TNF-α, IP-10, IL-2, IL-9, IL-17, IL-6, EGF, sCD40L, TGF-α, and GM-CSF) via multiplex assay MagPix (Luminex, USA) at Days 1, 42, and 140 after the first vaccine administration.

**Figure 3:**
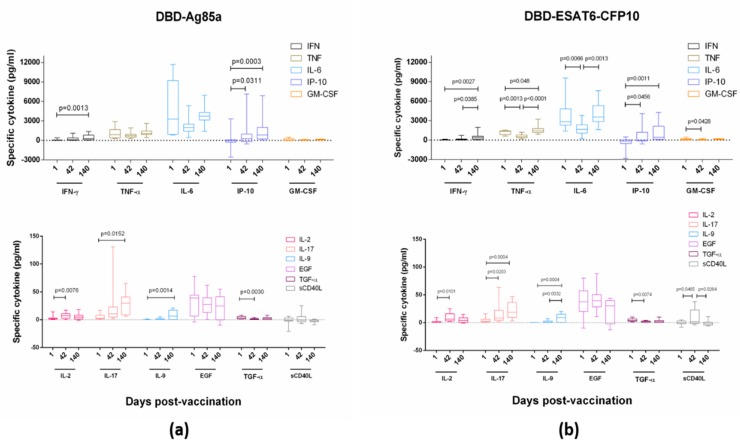
Vaccine-specific cytokine levels in Group 4. (**a**) Stimulation with DBD-Ag85a antigen; and (**b**) stimulation with DBD-ESAT6-CFP10 antigen. Blood cytokine levels specific to two vaccine proteins were measured at Days 1, 42, and 140 after stimulation by vaccine antigens. Lines, medians; boxes, IQT; whiskers, 5–95 percentiles. Baseline indicated with dotted line. Wilcoxon matched-pair signed rank test was used to detect differences between time points.

GamTBvac modified pre-existing cytokine responses (measured at baseline, Figure 3). DBD-Ag85a stimulated the secretion of IL-2 and TGF-α at Day 42 of observation, as well as IP-10, IL-17 and IL-9 during the whole period of observation. DBD-ESAT6-CFP10 induced IL-2, TGF-α, and GM-CSF at Day 42 and TNF-α, IP-10, IL-17, and IL-9 140 days postvaccination. No significant increase in IL-6, EGF, and sCD40L was observed.

#### 3.3.3. Antigen-Specific IgG Response

**Figure 4:**
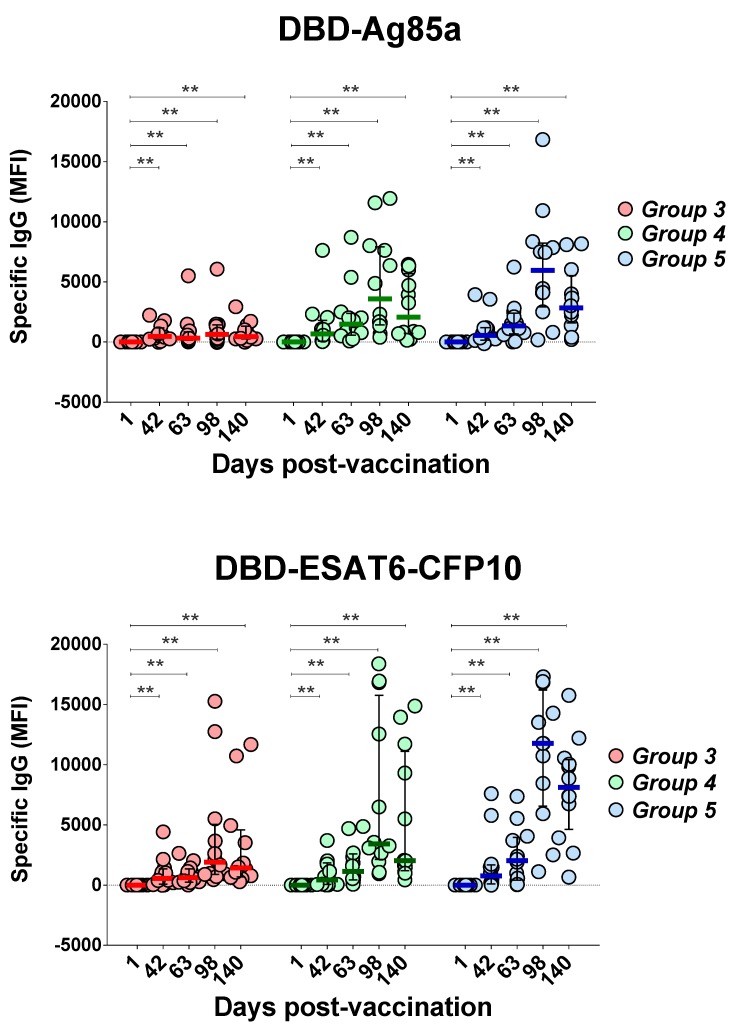
Changes of GamTBvac IgG antibody titers in three groups under investigation. IgG levels specific to two vaccine proteins were measured on five study days beginning with first day after first vaccination as baseline. Medians and IQT are shown. Baseline indicated with dotted line. Difference in medians was not statistically significant between groups (DBD-Ag85a overall *p* = 0.1985; DBD-ESAT6-CFP10 overall *p* = 0.6471). Wilcoxon matched-pair signed rank test was used to detect differences between time points in same group. ** *p* < 0.01.

Serum levels of IgG antibodies specific to DBD-Ag85a and DBD-ESAT6-CFP10, recombinant proteins of the GamTBvac candidate vaccine, were measured using MIA MagPix (Luminex, USA). Five time points were investigated: Days 1, 42, 63, 98, and 140 (Figure 4).

Both antigens, DBD-Ag85a and DBD-ESAT6-CFP10, stimulated the production of specific IgG antibodies in all groups, with an increase in antibody titer by Day 42 with an additional increase after the second vaccination with GamTBvac compared to the baseline titers. A dose-dependent effect was observed: in Group 3, an increase in the titer of IgG antibodies to DBD-Ag85a was significantly reduced comparing to the other two groups.

The contribution of individual antigens included into the candidate vaccine to the formation of IgG profiles was lower in general than that of fused molecules, and distributed as follows: CFP10, Ag85A, and DBD stimulated IgG production at Day 98 postvaccination, and later in Groups 4 and 5, while a significant reaction toward the ESAT6 antigen was only observed in Group 5 at Day 63 (*p* = 0.0401). In Group 3 (low vaccine dose), no significant increase in IgG titers was detected.

Our results propose that a second vaccination was essential for the formation of stable humoral immune response to GamTBvac antigens. After reimmunization at Day 67, IgG titers to both fusion antigens were maintained until the end observation at Day 140, which indicated the formation of stable memory that provided a humoral response to vaccine antigens. A comparison of antigen-specific IgG response between the groups showed higher antibody titers for the half and standard doses compared to the low dose. Differences between the half and standard doses were not significant.

## 4. Discussion

In the present Phase I clinical trial, the safety and preliminary immunogenicity of the candidate vaccine GamTBvac were investigated in previously BCG-vaccinated healthy adults. GamTBvac had an acceptable safety profile and was well-tolerated. In the course of the study, a significant difference in the number of volunteers with AEs among the placebo, single, and double administration schemes was established. At the same time, there was no correlation between the dose of the vaccine at the double-administration scheme and the number of volunteers with AEs: 10 (83.33%) volunteers in Group 3, 12 (100.00%) volunteers in Group 4, and 12 (100.00%) volunteers in Group 5. No serious AEs were reported, irrespective of the vaccine dose and time of administration. Two AEs considered to possibly be related to the vaccination were documented in the group with the highest dose of the vaccine introduced twice: an increase of C-reactive protein and body temperature. However, it was hardly possible to directly associate these AEs with vaccine administration. The vaccine mainly reported systemic events related to raised laboratory blood parameters, such as C-reactive protein, blood creatine phosphokinase, and red-blood-cell sedimentation-rate increase. In accordance with the protocol that the volunteers were followed, AEs were resolved spontaneously without the use of any concomitant therapy after 72 h. It can be concluded that the scheme with double administration of the vaccine in different doses approximately had the same safety profile.

**Figure 5:**
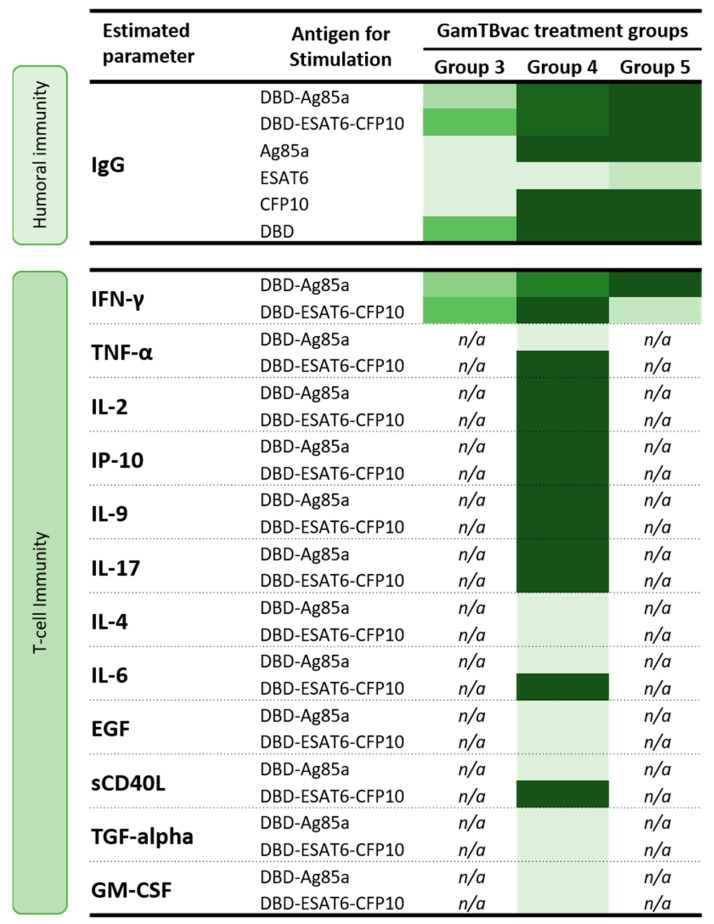
Vaccine-induced specific immune responses in three groups of volunteers assessed for immunogenicity. Heatmap: light-green, minimal values; dark-green, maximal values.

We showed that both antigens included in the vaccine (DBD-Ag85a and DBD-ESAT6-CFP10) induced specific immune responses. Vaccination was accompanied by the induction of both humoral (IgG antibody levels) and cellular immunity, expressed in IFN-γ increase, and a number of whole-blood lymphocyte-produced cytokine increase in response to in vitro stimulation with vaccine antigens. The effect was most pronounced in groups with double (half and high) administration of the vaccine (Groups 4 and 5). The summary of these findings is represented in Figure 5.

For tuberculosis, the core population providing protective immunity is considered to be a population of T-helper cells of the first type (Th1), producing IFN-γ, which is necessary for activation of macrophages and the development of an effective immune response toward intracellular infections [18,19]. The effect of IFN-γ on macrophages can be diverse, including phagocytosis stimulation and phagosome formation, stimulation of nitric oxide (II), reactive-oxygen-species synthesis, and expression increase of the MHC Class II by macrophages, which are necessary for the presentation of antigens to CD4 + T-lymphocytes [20,21].

Previously conducted testing of GamTBvac in murine and guinea-pig TB models showed that lymph-node cells were able to efficiently proliferate in response to antigens Ag85A and ESAT6-CFP10, while neither DBD domain alone nor adjuvant components without antigens were responsible for specific immunogenicity [9]. The current study confirmed that administration of GamTBvac induced durable ESAT6-CFP10- and Ag85a-specific T-cell responses by an increase in IFN-γ as was measured by the IFN-γ ELISA assay. It is worth noting that, in the case of the DBD-ESAT6-CFP10 antigen, in the group with the full vaccine dose, the levels of IFN-γ production after blood stimulation were higher compared to the half and low doses. However, a significant drop in cytokine production was detected at the last observation point (140 days), while, for the groups of half and low doses, IFN-γ levels continued to increase. It can be assumed that this decrease was due to the rapid death of memory-cell populations in volunteers who received a full dose of the vaccine, which may determine the lack of development of long-term memory with the investigated vaccination scheme. Thus, half-dose vaccination is preferable for GamTBvac vaccine formulation for subsequent studies.

The importance of production activation of IL-2 and TNF-α cytokines for the formation of protective immunity against tuberculosis vaccines was noted and previously actively discussed [19,22,23,24]. An increase in the expression of three cytokines, IL-2, IFN-γ, and TNF-α, when stimulated with DBD-Ag85a or DBD-ESAT6-CFP10, indicated the development of antigen specificity of immune response after GamTBvac vaccination and suggests the protective efficiency of candidate vaccine.

In this study, the three vaccine groups had a comparable dose-dependent increase in serum vaccine-specific IgG after the first vaccination. The role of these antibody responses is unclear and needs to be further investigated. BCG can induce antibodies, although only at modest levels. However, it showed to be enough to have antimycobacterial effects [25,26,27]. It should be mentioned that, for H1/IC31 and H56/IC31 subunit vaccines, a limited antibody response, about 10% in MTB-naïve individuals, but a significant response in MTB-infected participants (60%), were observed after two vaccinations [28,29,30], while a third vaccination with H56/IC31 raised the response to 60% in MTB-naïve participants [31]. Vaccines with antibody-boosting responses, primed by the BCG vaccine, could be part of strategies for TB vaccine development. Unfortunately, the disappointing fact that is being accepted by wide expert community now is that there are no validated immunological markers—correlates of protection (COP)—that can be used to guide TB vaccine-candidate studies [4]. On the one hand, the lack of an efficient vaccines, protective in human or relevant animal models, does not allow finding proper COP. On the other hand, the lack of relevant immunity markers makes it difficult to develop a protective vaccine. In this regard, broader vaccine effects analysis, including mucosal immunity induction, antibody responses, and nonconventional T-cells responses, might also help to explain some protective conditions [2], such as BCG vaccination [32], latent infection [33,34], and sterile immunity mediated by antibodies [35]. Thus, the registration of TB vaccine candidates after the relevant safety evaluations might be a straightforward way to speed up initiations of post-registration studies of efficiency in specific but efficiently large groups of people. Such an approach would allow significantly reducing the cost of studies on thousands of people. It would also stimulate researchers to improve protective compositions instead of studying the immunogenicity of nonprotective candidates.

## 5. Conclusions

Here, we present the results of the first-in-human trial of recombinant subunit tuberculosis vaccine GamTBvac in a Phase I study. Safety and immunogenicity were estimated in an open-label clinical trial on 60 MTB-uninfected BCG-vaccinated volunteers. The vaccine was safe and well-tolerated in different doses of the used antigens. Three different doses of the vaccine with a double-immunization scheme were assessed for immunogenicity, among which a half dose showed the most promising effects. A half dosage allowed achieving an early (beginning from Day 42 of observation), stable (up to Day 140 of observation) and high (comparable to the full-dose level) immune response to all antigenic components of the vaccine, as well as inducing the development of a complex immune response. The overall evaluation of the safety of the GamTBvac candidate vaccine allowed the initiation of further clinical trials for it (ClinicalTrials.gov identifier: NCT03878004 [36]) with the perspective of further registration for use in healthy BCG-vaccinated adults and initiation of a post-registration observation study of populational effectiveness. The second phase includes safety evaluation and immunogenicity in 180 healthy BCG-vaccinated volunteers randomly allocated to groups of placebos and half-dose GamTBvac in a 1:3 ratio. A clear demonstration of safety would help to persuade Ministry of Health experts to register use of GamTBvac on healthy BCG-vaccinated adults and initiate further studies of populational effectiveness.

## 6. Patents

According to the results of this work, the following patent was issued: RU 2 665 817 C1 RECOMBINANT TUBERCULOUS VACCINE AND ADJUVANT FOR IT, Tkachuk Artem Petrovich (RU), Gushchin Vladimir Alekseevich (RU), Manujlov Viktor Aleksandrovich (RU), Gudov Vladimir Petrovich (RU), Lunin Vladimir Glebovich (RU), and Gintsburg Aleksandr Leonidovich (RU)

## Figures and Tables

**Table 1:** Study groups.

Title	Antigens	CpG2216	DEAE-Dextran 500	Dextran 500	Vaccination
Group 1 (PBS Placebo)	no	no	no	no	Single
Group 2	6.25 μg DBD-ESAT6-CFP10 6.25 μg DBD-Ag85a	37.5 μg	125 μg	2.5 mg	Single
Group 3	6.25 μg DBD-ESAT6-CFP10 6.25 μg DBD-Ag85a	37.5 μg	125 μg	2.5 mg	Double
Group 4	12.5 μg DBD-ESAT6-CFP10 12.5 μg DBD-Ag85a	75 μg	250 μg	5 mg	Double
Group 5	25.0 μg DBD-ESAT6-CFP10 25.0 μg DBD-Ag85a	150 μg	500 μg	10 mg	Double

**Table 2:** Demographics of enrolled participants. The difference between groups was defined using one-way ANOVA with Tukey’s test for multiple comparisons (*p* < 0.05).

Title	Group 1 (*n* = 12)	Group 2 (*n* = 12)	Group 3 (*n* = 12)	Group 4 (*n* = 12)	Group 5 (*n* = 12)
**Age**					
Median age, years (min–max)	28.5 (23.0–43.0)	25.5 (20.0–47.0)	39.5 (24.0–49.0)	22.0 (20.0–47.0)	22.5 (19.0–45.0)
**Sex**					
Male, *n* (%)	3 (25.00%)	8 (66.67%)	1 (8.33%)	2 (16.67%)	3 (25.00%)
**Ethnicity**					
European	12 (100.00%)	12 (100.00%)	12 (100.00%)	12 (100.00%)	12 (100.00%)
Median body mass index, kg/m^2^ (min–max)	22.80 (18.90–27.60)	21.00 (19.20–27.00)	25.55 (19.50–28.70)	21.50 (20.30–28.40)	23.08 (19.60–28.70)

**Table 3:** Related adverse events (AE). Local and systemic adverse events that occurred following vaccination are shown. Frequency was calculated as number with AEs/total number of participants in group, %.

Title	Group 1 (*n* = 12)	Group 2 *n* = 12)	Group 3 (*n* = 12)	Group 4 (*n* = 12)	Group 5 (*n* = 12)
Participants with at least one AE, *n* (%)	0 (0.00%)	6 (50.00%)	10 (83.33%)	12 (100.00%)	12 (100.00%)
**Local-injection-site AEs (number with AEs after first and second immunization (% of participants with AE)**					
Erythema	0 (0%)	6 (50%) ^1^	8/0 (66.67/0%) ^1,2^	12/0 (100/0%) ^1^	10/2 (83/17%) ^1^
**Systemic AEs (number with AEs/total number of participants in group, %)**					
Gastrointestinal disorders					
Dyspepsia	0 (0.0%)	0 (0.0%)	0 (0.0%)	1 (8.3%)	0 (0.0%)
General disorders					
Body-temperature increase	0 (0.0%)	0 (0.0%)	0 (0.0%)	0 (0.0%)	1 (8.3%)
Laboratory abnormalities					
Red-blood-cell sedimentation-rate increase	0 (0.0%)	0 (0.0%)	0 (0.0%)	1 (8.3%)	1 (8.3%)
C-reactive protein increase	0 (0.0%)	0 (0.0%)	1 (8.3%)	3 (25.0%)	1 (8.3%)
Alanine aminotransferase increase	0 (0.0%)	0 (0.0%)	0 (0.0%)	1 (8.3%)	0 (0.0%)
Aspartate aminotransferase increase	0 (0.0%)	0 (0.0%)	0 (0.0%)	1 (8.3%)	0 (0.0%)
Blood creatine phosphokinase increase	0 (0.0%)	0 (0.0%)	0 (0.0%)	2 (16.7%)	2 (16.7%)
Metabolism and nutrition disorders					
Hypercholesterolemia	0 (0.0%)	0 (0.0%)	1 (8.3%)	0 (0.0%)	0 (0.0%)
Total number of AEs in group (*n* = 54)	0 (0.0%)	6 (11.1%)	10 (18.5%)	21 (38.9%)	17 (31.5%)

^1^ All cases of hyperemia were less than 8 cm in diameter, appeared within the first 24 h, and disappeared within 72 h postvaccination (slight swelling and soreness at the injection site were observed). ^2^ Primary/repeated administration.
